# Constraints on genes shape long-term conservation of macro-synteny in metazoan genomes

**DOI:** 10.1186/1471-2105-12-S9-S11

**Published:** 2011-10-05

**Authors:** Jie Lv, Paul Havlak, Nicholas H Putnam

**Affiliations:** 1 Department of Ecology and Evolutionary Biology, Rice University, Houston TX 77098, USA

## Abstract

**Background:**

Many metazoan genomes conserve chromosome-scale gene linkage relationships (“macro-synteny”) from the common ancestor of multicellular animal life [1-4], but the biological explanation for this conservation is still unknown. Double cut and join (DCJ) is a simple, well-studied model of neutral genome evolution amenable to both simulation and mathematical analysis [5], but as we show here, it is not sufficent to explain long-term macro-synteny conservation.

**Results:**

We examine a family of simple (one-parameter) extensions of DCJ to identify models and choices of parameters consistent with the levels of macro- and micro-synteny conservation observed among animal genomes. Our software implements a flexible strategy for incorporating genomic context into the DCJ model to incorporate various types of genomic context (“DCJ-[C]”), and is available as open source software from http://github.com/putnamlab/dcj-c.

**Conclusions:**

A simple model of genome evolution, in which DCJ moves are allowed only if they maintain chromosomal linkage among a set of constrained genes, can simultaneously account for the level of macro-synteny conservation and for correlated conservation among multiple pairs of species. Simulations under this model indicate that a constraint on approximately 7% of metazoan genes is sufficient to constrain genome rearrangement to an average rate of 25 inversions and 1.7 translocations per million years.

## Background

### Macro-synteny conservation

Recent genome sequencing efforts have dramatically expanded the sampling of metazoan diversity represented among assembled genomes. One unexpected result of comparing these genomes is that their chromosome-scale organization is largely conserved from the last common ancestor of metazoans in members of multiple phyla. The genomes of sponges, cnidarians, placozoans, and chordates all show extensive conservation of chromosome-scale linkage (or macro-synteny) among genes [1-4].

**Figure 1:**
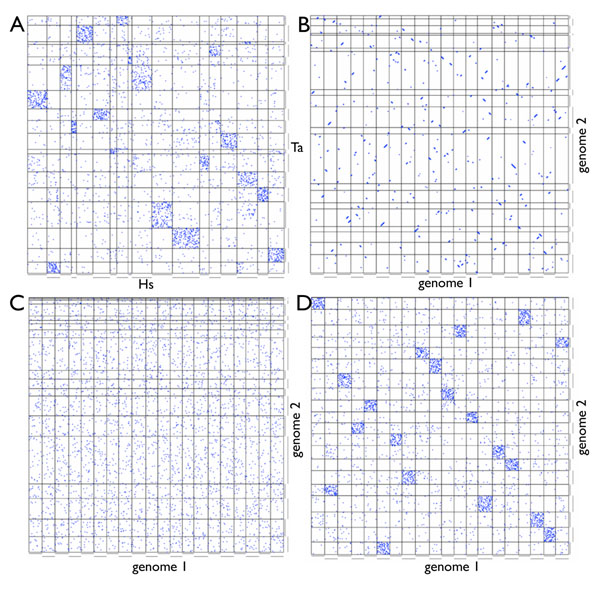
**Whole-genome gene order comparison (“dot plots”)** for (a) Observed: human (Hs) vs. placozoan (Ta). (b) Starting (genome 1) and ending (genome 2) genomes of DCJ simulations matching conserved macro-synteny ( *p* = *p*_HsTa_ ). (c) DCJ simulation matching conserved micro-synteny: ( *s* = *s*_HsTa_ ). (d) DCJ-DS simulation matching both conserved micro- and macro-synteny ( *mu* = 0.07, *n* = 30,000 ).

Only a handful of genome projects (and only the human genome among those examined in this work) have received sufficient depth of sequencing, long-range clone-end sequencing, and map construction for their longest reconstructed pieces (called “scaffolds”) to approach the length of whole chromosomes. However, indirect methods have been developed [1,2] to infer chromosome-scale linkage from orthologous genes shared between scaffolds of different draft genome assemblies. We apply those methods here to partition the scaffolds (or chromosome segments, in the case of the human genome) of five metazoan genomes by biclustering. In the resulting partitioning, the scaffolds in each group share a distinct distribution of orthologs across the groups of other genomes. This pattern is clearly visible in the human-piacozoan “dot plot” of Figure 1a.

This pattern has been interpreted as indicating that each such group of scaffolds corresponds to an ancestral chromosome (predating recent genome rearrangements), and the groups are therefore referred to as “putative ancestral linkage groups” (PALs). This interpretation is bolstered by statistical tests, and one PAL of *Branchiostoma floridae* was interrogated by physical mapping and found to correspond to a single chromosome [2]. While this imprint of the ancestral metazoan chromosomes clearly persists in the genomes analyzed here which have been diverging for over half a billion years, it has been mostly or completely lost in the genomes of all sequenced arthropods, nematodes, and tunicates [2,6].

Several biological mechanisms could explain the observed conservation, including a low average rate of germ-line mutations involving inter-chromosomal rearrangement, and low rate of fixation of these mutations because they disrupt gene regulatory interactions. We set out to find simple, concrete models of genome evolution that can explain such synteny conservation, and can be used to generate simulated null distributions for testing hypotheses about genome evolution.

### Extending the DCJ model with constraints

DC J is a generic genome rearrangement operation determined by (1) selecting a pair of points at which the genome is cut, and (2) reconnecting the resulting new ends to effect an inversion, a reciprocal translocation, or the excision, insertion, fission or fusion of circular fragments. All gene-conserving moves of genome rearrangement can be constructed from DCJ operations. Efficient algorithms exist for computing the minimum number of DCJ operations required to transform one genome into another [5,7]. DCJ has also previously been used to study the behavior of genomes evolving under a stochastic model in which DCJ moves are selected by choosing cut points uniformly at random across the genome, and with constraints imposed on move choices designed to more closely match observations of real genomes [8,9]. Stochastic DCJ with cut points selected at random (hereafter referred to as the DCJ model) imposes a fixed relationship between the rate of decay of micro- and macro-synteny, and as we show below, it cannot account for the long-term conservation of macro-synteny relationships.

We have examined four models which build on DCJ, each through the addition of a single adjustable parameter which affects the relative frequency and/or size of intra- and inter- chromosomal rearrangement events by imposing various constraints on move selection. In each case, candidate DCJ moves are proposed at random, and either carried out or rejected according to the rules of the model. We considered the following models:

**DCJ-max***_L_***:** Under this model, a maximum rearrangement length (*L_max_*) is imposed on inversions, excisions and translocations.

**DCJ-max***_T_***:** This model is similar to DCJ-max*_L_* but the length restrictions are imposed only on the inter-chromosomal operations, and not on inversions.

**DCJ-p**_fix_**:** In this model, proposed inter-chromosomal rearrangements are independently accepted with probability *p_f_ix*, and otherwise rejected, reducing their frequency by not their size.

**Figure 2:**
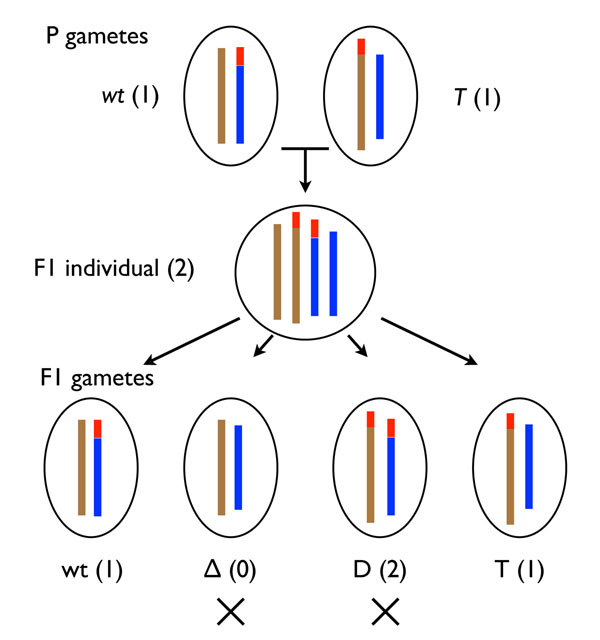
**Structural polymorphisms result in gene dosage changes.** Top row: haploid gametes with chromosomes represented as colored vertical bars, with wild genotype (*wt*) and mutated genotype carrying a single translocation (*T*); middle row: heterozygous diploid individual; bottom row: gametes produced at the next generation, half of which have altered dosage: the translocated segment (red color) is either deleted (Δ), or duplicated (*D*)*.* Numbers in parentheses indicate the number of copies of the translocated segment.

**Figure 3:**
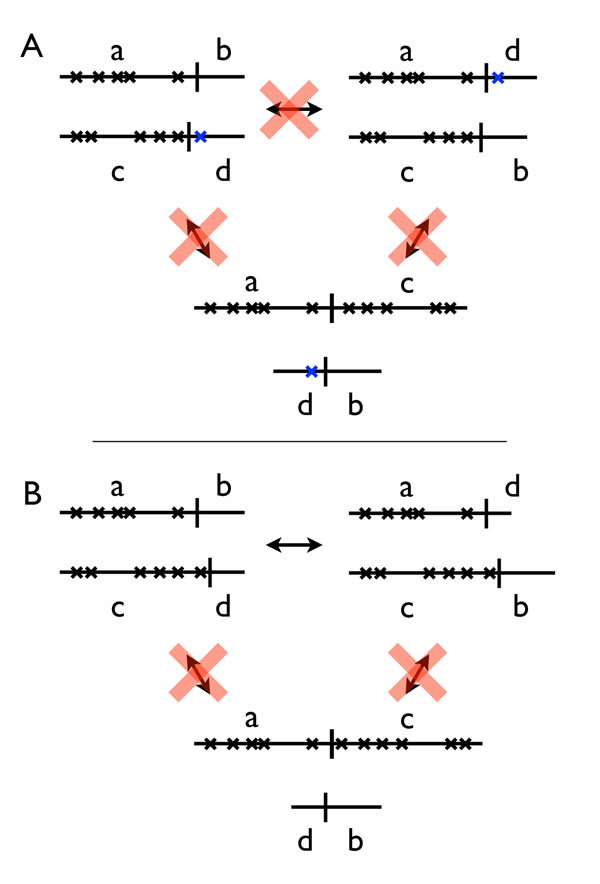
**DCJ-DS model.** The four chromosomal fragments produced by a pair of cuts on different chromosomes can be rejoined in two ways. Inter-chromosomal rearrangement that would change the chromosomal-scale connectivity of sensitive genes (x’s) are rejected. Rearrangements are accepted only when one side of each breakpoint is free of sensitive genes both before and after the move. A: Illegal cuts. Connectivity of sensitive genes would be broken in any of these moves. B: Illegal rejoins. Sensitive genes from different chromosomes would be rejoined (or split) in the rejected moves.

**DCJ-DS:** A fixed fraction of genes *µ* are flagged as “sensitive”, and operations that would alter the partitioning of these genes among chromosomes are rejected. DCJ-DS was conceived to study constraints on genome structure evolution arising from dosage-sensitive genes (Figure 2); *i.e.*, genes producing a phenotypic effect when their copy number in a diploid genome deviates from the normal complement of two. Dosage sensitivity has been shown to play a role in determining the long-term fate of genes created by whole genome duplication [10]. A new mutation moving a dosage-sensitive gene from one chromosome to another is unlikely to be fixed in a diploid population, because when crossed with the un-rearranged genotype it leads to gametes with zero and two copies of the translocated genes, in addition to those with one copy. This leads to underdominant selection against such rearrangements, as illustrated in Figure 2. Figure 3 illustrates examples of the operation of the constraint.

Other types of constraint that restrict the movement between chromosomes of a fixed subset of genes may have similar or indistinguishable effects when realized in such a simplified model. Examples of such subsets of constrained genes could, for example, include genes under the control of long-range cis-regulatory elements.

## Results

### Simulations of genome evolution

Our open-source Python implementation of these methods enables simulation of genome evolution under a family of models based on DCJ. These extend the double cut and join paradigm by rejection of moves based on various types of genomic context, and we refer to them collectively as “DCJ-[C]”. Our software includes a modified binary search tree with “reverse” flags and subtree summaries on nodes so that all the information necessary to carry out DCJ-[C] operations, such as counting “sensitive” genes on any fragment, can be completed in O(log N) time. [11-13] Although we do not enforce balanced binary trees for a strict bound on performance, we found chromosome gene trees to remain O(log N) height on average, with correspondingly fast running time. (Data not shown.) The software can be downloaded from http://github.com/putnamlab/dcj-c.

We define two summary statistics: *s_ab_* and *p_ab_*, which measure the conservation rate of micro- and macro-synteny respectively in a pairwise comparison of genomes *a* and *b. s_ab_* is equal to the fraction of gene adjacencies in *a* which are also present in the orthologous genes of *b. p_ab_* is the fraction of genes in genome *a* which have a conserved chromosomal context in *b.*

We focus on five metazoan genomes representing anciently-diverged metazoan groups that have been shown previously to exhibit extensive macro-synteny conservation, and for which PALs have previously been inferred: *Homo sapiens* (human) [14], *Branchiostoma floridae* (lancelet) [2], *Nematostella vectensis* (sea anemone) [1], *Trichoplax adherens* (placozoan) [3] and *Amphimedon queenslandica* (sponge) [4]. These genomes have pairwise values of *p* ranging from 35% to 58%, and of *s* ranging from 0.4% to 2.3% (Table 1). All the models considered reduce to the DCJ model for some choice of the added parameter. DCJ does not predict the levels of micro- and macro- synteny observed among these genomes. In simulation, when the level of macro-synteny conservation falls to ≈ 50%, the average value of *s* is ≈ 90%(Figure 1b). At longer simulated evolutionary times, *p* falls to its saturation level of 1/*c*, where *c* is the number of chromosomes, by the time *s* approaches the range observed in the metazoan data (Figure 1c; Table 1).

**Table 1:** 

Genomes	markers	*p*	*s*	*n*	*µ* (%)	*n_t_*
Hs-Bf	4408	.58	.0218	26441	7.27	1712
Hs-Nv	3451	.45	.0038	49650	7.93	2931
Hs-Ta	3557	.51	.0138	30122	6.96	2115
Hs-Aq	2400	.35	.0038	49550	6.89	3328
Bf-Nv	3972	.51	.0055	42970	8.17	2431
Bf-Ta	3970	.59	.0229	25492	7.39	1637
Bf-Aq	2690	.42	.0082	35917	6.62	2602
Nv-Ta	2664	.39	.0141	30652	7.75	1868
Nv-Aq	3972	.57	.0049	43878	6.90	3092
Ta-Aq	2953	.44	.0152	27394	5.96	2232

**Figure 4:**
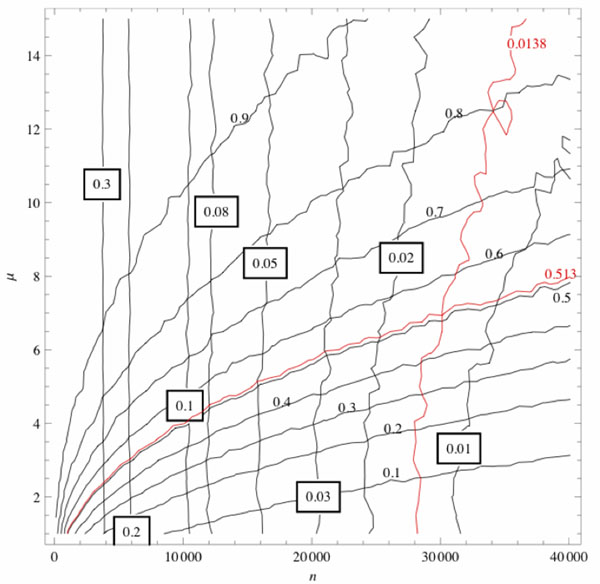
**Dependence of *p* and *s* (macro- and micro-synteny conservation rates) on *n* and *µ* (number of rearrangements and fraction of dosage sensitive genes).** Boxed and un-boxed numbers label contours of equal *s* and *p* respectively. Red contour lines have values equal to *p*_HsTa_ and *s*_HsTa_. Their crossing point indicates that observed levels of synteny conservation between human and placozoan genomes can be obtained under the DCJ-DS model when *µ* ≈ 7% and *n* ≈ 30000.

For each constrained model, we explored the dependence of the mean values of *s* and *p* on the number of rearrangements and the added model parameter. Figure 4 shows the dependence of the average values of *s* and *p* when comparing starting and ending genomes of DCJ-DS simulation runs as functions of *n*, the number of accepted moves and *µ*, the fraction of dosage-sensitive genes. The rate of decay of micro-synteny with *n* depends only weakly on *µ*, while macro-synteny decays much more slowly with increased *µ.*

### Comparisons to genome data

We fit each model to the Human-Trichoplax divergence, which exhibits typical levels of *p* and *s*, and both genome have high quality assemblies. Three of the models can simultaneously account for the observed levels of micro- and macro-synteny observed in pairwise genome comparisons (Figure 4); only the DCJ-Lmax fails in this respect. While restricting the size of all rearrangements does slow the decay of macro-synteny due to lower frequency and size of inter-chromosomal rearrangements, the loss of micro-synteny is also slowed when inversion size is limited [15]. The best-fitting parameter values of each model to the human-placozoan comparison are shown in Table 2.

**Table 2:** 

Model name	*n* *Hs - Ta*	parameter name	value
DCJ-max*_L_*	*-*	L_max_	-
DCJ-max*_T_*	28948	T_max_	20
DCJ-p_fix_	34532	p_fix_	0.0000267
DCJ-DS	30122	p	0.0696

### Multi-species comparison

To further discriminate among the models and compare them to the genome data, we examined their behavior in a multi-species comparison. Because a specific subset of genes is strictly constrained to remain on their starting chromosome in the DCJ-DS model, this model implies correlations in the fates of orthologous genes in lineages after they diverge. In the real data, we found 1144 single-copy gene families present in all five genomes, of which 298 showed conserved macro-synteny in all five genomes. This is more than what one might expect based on a simple model of independent gene movement (, although this calculation does not take into account correlations that could be induced by the discrete nature of the rearrangement process, shared ancestry, or variation in evolutionary rates. Table 3 lists symbols and abbreviations.)

**Table 3:** List of symbols

*n*	Number of genome rearrangements
*N*	Number of shared markers (genes) used in a genome comparison
*n_t_*	Number of interchromosomal genome rearrangements
*µ*	The fraction of dosage sensitive genes
S_ab_	The fraction of conserved gene adjacencies (micro-synteny)
p_ab_	The fraction of genes constributing to conserved macro-synteny
c	Number of chromosomes
*p_fix_*	Probability with which proposed interchromosomal rearrangements are accepted in the DCJ-p_fix_ model
Hs	*Homo sapiens;* human
Bf	*Branchiostoma floridae;* lancelet
Nv	*Nematostella vectensis;* sea anemone
Ta	*Trichoplax adhaerens;* placozoan
Aq	*Amphimedon queenslandica;* sea sponge

DCJ	Double cut and join
DCJ-[C]	Double cut and join, with context-dependent constraints
DCJ-DS	Double cut and join, with dosage-sensitive constraint
DCJ-max*_L_*	Double cut and join, with maximum rearrangement size
DCJ-max*_T_*	Double cut and join, with maximum translocation size
DCJ-p_fix_	Double cut and join, with translocations made rare
PAL	Putative ancestral linkage group

We measured multi-species conserved macro-synteny for each of the three successful models by sampling simulated evolutionary histories of five species in a star-shaped phylogeny, with the same mean pairwise micro- and macro-synteny conservation rates as the real data. For each simulation we selected 1144 marker genes and counted how many exhibited five-way conserved macro-synteny. The DCJ-Tmax and DCJ-p_fix_ models, which treat all genes equally, matched the prediction of independent gene movement, while the DCJ-DS model showed a level consistent with that found in the real data.

**Figure 5:**
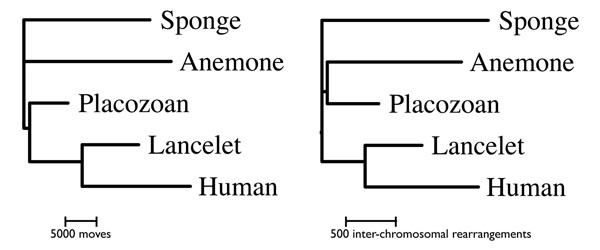
**Neighbor-joining trees.** Based on the total number of rearrangements (left) and the number of inter-chromosomal rearrangements (right) indicated by the best fit to the DCJ-DS model.

**Figure 6:**
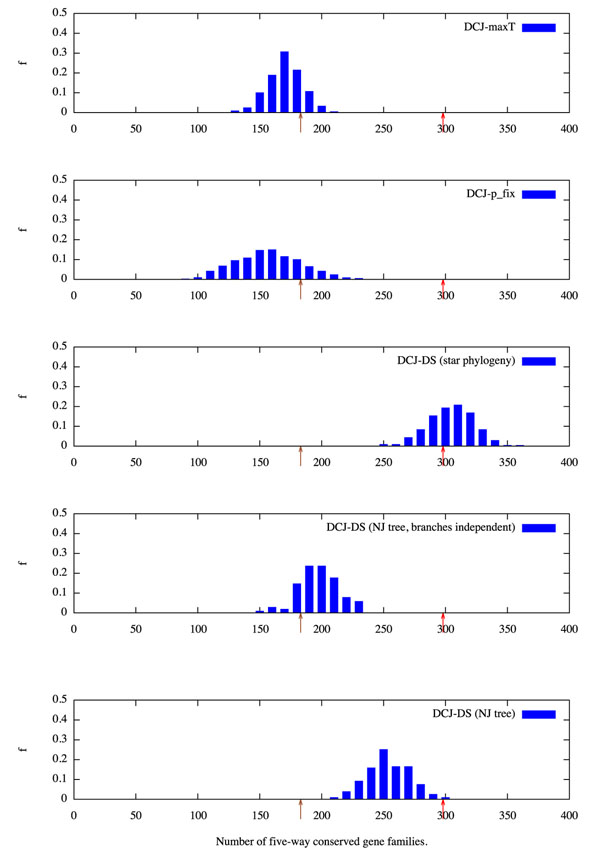
**Multi-species conserved macro-synteny.** Normalized frequency distributions of the number of gene families (out of a total of 1144) conserved in all five leaves of the tree for various models. The observed number in the real data (298) is indicated with a red arrow, and the expectation of a simple model of independent gene movement (183) with a brown arrow.

We wished to further assess the impact of these estimates of variation in branch length, shared evolutionary history (*i.e.* a resolved, rather than star-shaped tree), and the move size and frequency distributions of the DCJ-DS model. Therefore we estimated the rearrangement distance *n* and fraction of marked genes *µ* for each pairing of five metazoan genomes which have previously been shown to conserve ancient macro-synteny relationships (Table 1). We then applied the neighbor-joining method to construct a distance-based phylogenetic tree (Figure 5). We simulated the evolution of the genomes under the DCJ-DS model across this tree multiple times, and measured the multi-species conservation rate under two conditions. In the first condition, the same set of genes was marked as dosage sensitive across the entire tree in each simulation run. In the second condition, marked genes were chosen independently for each branch in the tree, preserving the rearrangement dynamics of the DCJ model, but not the correlations among lineages. The distribution of conservation rates is shown in (Figure 6).

## Discussion

Three of the four models we considered can account for the pattern of synteny conservation observed between pairs of metazoan genomes, but only the DCJ-DS model explains the high observed level of multiple-species macro-synteny conservation. This suggests that constraints on a specific subset of genes, arising from some link biological function are responsible for shaping the long term evolution of metazoan genome organization.

DCJ-DS is a biologically motivated extension of the DCJ model, adding a single new parameter, (*µ*)*:* the fraction of genes that are constrained against movement between chromosomes, modeling the effect of dosage-sensitive selection. The simulation results presented here show that this model provides a plausible and sufficient explanation for the observed large-scale patterns of genome organization that we examined here. In particular, such a constraint acting on only ≈ 7% of genes is sufficient.

The models considered here are, like DCJ, highly simplified models of genome rearrangement, ignoring many important aspects of genome evolution, such as gene duplication and loss, chromosome fission and fusion, variation in the propensity for rearrangement across the genome, turnover in the population of dosage-sensitive genes, and genetic drift. The DCJ-[C] framework may be useful in future tests of the effects that these factors have had on the evolution of genome organization as more genomes are assembled. The software we have developed is well-suited to the implementation of other extensions of the DCJ model, and we have made our code available to the community for this purpose. For example, DCJ moves could be restricted based on the inclusion or non-inclusion of other features such as centromeres, the topologies of the chromosomes affected or produced, and others.

The evolutionary dynamics of genome rearrangement in nature are unlikely to match any of the one-parameter models considered here in detail. More richly parametrized models can allow the frequencies of various rearrangement types, and the size distributions of rearranged fragments all to vary independently. But because one-parameter models are easy to understand, implement, estimate, and compare with one another, when they can account for the data, we contend they are worth considering. The neighbor-joining analysis of rearrangement distances (Figure 5) groups human and lancelet together, but does not clearly resolve any other relationships. The short branch leading to the Placozoan genome is consistent with previous observations of the conserved nature of *Trichoplax* genome organization [3]. The long branch leading to human is likely due in some part to the fact that DCJ-DS ignores the scrambling effect of the two rounds of whole genome duplication, followed by extensive gene loss in the vertebrate common ancestor. Using divergence time estimates based on a combination of molecular and fossil data [16], the total lengths of the trees indicate mean rates of 25 rearrangements per million years. This rate is intermediate to rates estimated within vertebrates (0.1 - 0.4 breaks / million years [17]), nematodes (48 / million years [18]) and flies (17-21 / million years [19] ). However, rearrangement rate comparisons between studies must be interpreted cautiously because estimated rates are dependent on the density of markers used. On average, inversions occurred approximately 15 times more frequently in our DCJ-DS simulations with *µ* = 7% than inter-chromosomal rearrangements.

The genomes selected for this analysis show extensive macro-synteny conservation from the common ancestor of metazoans, but what about those that are known to conserve it to a much lesser extent (such as *Ciona intestinalis*) or to have lost it entirely (such as *Drosophila melanogaster* and other arthropods, *Caenorhabditis elegans* and other nematodes, and *Oikopleura dioica*) [*2*]? These losses may be due to a faster “molecular clock” on these branches, increased rates of chromosome fusion (which are not allowed in DCJ-DS among chromosomes containing sensitive genes), or a reduction in the DS barrier. Additional genome sequences and further comparative analysis may be able to distinguish these possible explanations.

## Conclusions

This study shows that the DCJ model of genome evolution can be extended to generate simple models sufficient to explain the observed levels of micro- and macro-synteny conservation, by directly limiting the size and/or frequency of inter-chromosomal rearrangements, or by constraining the movement between chromosomes of a small fraction of genes.

Of the models we examined, only the DCJ-DS model, which singles out a class of genes for constraint, could account for the observed levels of correlation in gene fates across the tree. We argue that it is unlikely that any model which treats all genes symmetrically can account for this level of correlation, and that this result strongly suggests a causal link of some kind between gene function and the long-time scale evolution of metazoan genome organization at the chromosome scale.

We propose a simple model for such a causal link in which a fraction of dosage-sensitive genes cannot move between chromosomes because mutations which would carry them to a new chromosome are subject to underdominant selection, preventing their spread in a population. These results do not rule out other causal mechanisms, such as a fraction of genes on each chromosome which are constrained from moving by shared regulatory elements. As the quantity and quality of genome sequence and functional annotation data increases, it may soon be possible to distinguish these hypotheses through comparative genomic analysis and modeling.

## Methods

### Genome comparisons

We used a modified version of a distance-based, species phylogeny-guided gene ortholog clustering method that has been previously described [1,2] (Havlak et al, unpublished). To avoid the complications of gene gain and loss, we restricted each pairwise analysis of genomes to inferred one-to-one gene ortholog pairs. We pre-clustered the scaffolds (or chromosome segments, in the case of human) into PALs as previously described. [1-4]

To assign PAL homology relationships in pairwise comparisons of real and simulated genomes, we compute a z-score for each pair of PALs , where , and  are the observed number, expected number and expected variance in the number respectively of orthologous markers shared by *a* and *b* under a binomial approximation of the number of orthologs at saturation [1]. Each PAL is considered homologous to the PAL with which it has its highest z-score in the other genome.

### Simulation

In all simulations, the genome is initialized with 20 linear chromosomes, and a total of 20,000 genes. DCJ moves are proposed and either accepted or rejected according to the rules of each DCJ-[C] model until the desired number of moves (*n*) have been accepted and applied.

In the DCJ-DS model each gene is independently marked “sensitive” at random, with probability *µ.* Moves are rejected if they would result in a change in chromosome-scale linkage relationships among sensitive genes. This constraint means that throughout the simulation, the partitioning of sensitive genes by chromosome stays unchanged (Figure 3).

In the DCJ-max*_T_* model translocation operations are allowed only if one pair of exchanged fragments are both smaller than a threshold size T_max_. For example, the translocation illustrated in the top line of Figure 3b, in which two fragment *ab* and *cd* become *ad* and *cb*, would be allowed if *either* (*a* and *c*) *or* (*d* and *b*) are both smaller than or equal to T_max_. Similarly, excisions are allowed only if the excised fragment is not longer than T_max_. All other moves are accepted.

The DCJ-max*_L_* model imposes the constraints of DCJ-max*_T_*, and also rejects inversions if the inverted fragment is longer than *L_max_.*

In the DCJ-p_fix_ model, proposed translocations and excisions are accepted based on the outcome of a Bernoulli trial with success probablity *p_fix_*.

### Comparison of simulations to genomes

In order to compare the model to simulations, we define two statistical summaries of a pairwise comparison of genomes: *s*, the fraction of conserved marker gene adjacencies (*s*); and *p*, the fraction of marker genes with conserved PAL context. To compute *p*, we count the fraction of orthologous gene pairs (*i*, *j*) which reside on homologous PALs. In graphical terms, *p* measures the fraction of blue dots in the dense boxes of the genome comparison dot plots like those is Figures 1 a and d.

### Dealing with differing resolution in the pairwise comparisons

When simulation runs are compared to a real datasets, the statistics (*p*) and (*s*) are computed only on a set of “marker genes”, a random subset of the modeled genes selected to match the number of marker genes in the data for each comparison (Table 1). Because the DCJ-DS model is time-reversible, comparisons of simulated ancestor and descendant genomes are equivalent to comparisons of the leaves of two-taxon trees.

### Model parameter estimation and tree construction

Best-fit model parameters were estimated by numerically minimizing the sum of the squares of the normalized deviations between simulation runs and data of *s* and *p*; , where  are the means and standard deviations of the statistical summaries of ten simulation runs. We did this in two phases: in the first, we optimized *Χ*^2^ as a function of *n* and *µ* by successive one-dimensional minimization with respect to these two variables using Brent’s method. Because this method does not account for noise in the values of *s* and *p* obtained by averaging over a small number of simulation runs, we ran additional simulations to calculate *s*(*n*, *µ*) and *p*(*n*, *µ*) on a 7x7 grid in a region spanning ± 15% of the optimal values obtained in phase one. We fit a parabolic function in the neighborhood of this minimum using the Mathematica software package [20], and report the location of these minima as the best-fitting values of (*n*, *µ*)*.*

We built the distance-based phylogenetic tree with the neighbor-joining method [21] based on the pair-wise distances *n* listed in Table 1, as implemented in version 3.68 of the program *neighbor* of the PHYLIP package [22].

### Multiple-species macro-synteny comparison and simulations

In the real data we found 1144 gene ortholog groups represented exactly once in the PALs of the five genomes examined here. 298 of them are in conserved macro-synteny for all pairwise comparisons among the five genomes.

For the DCJ-max*_T_* and DCJ-max*_L_* models, to simulate evolution on star-shaped phylogenetic trees (with pairwise divergence *n* between leaves), we created a set of 100 simulation realizations of length *n*/2, all starting from the same gene order, with different random number seeds. We sampled five of them at a time without replacement, selected a common set of 1144 marker genes, and counted the number that remained in homologous chromosomes along all five simulated branches.

To simulate evolution on a star-shaped tree under the DCJ-DS model, five simulation runs were carried out from the same starting gene order and the same choice of sensitive genes, but with different random number seeds. To simulate evolution under DCJ-DS on the NJ trees, simulations were carried out along each branch, one set of simulations maintaining a fixed choice of marked genes, the other using an independent choice along each branch.

## Competing interests

The authors declare that they have no competing interests.

## Authors' contributions

NHP, PH and JL conceived the research. NHP, JL and PH wrote the software. JL carried out the simulations and genome comparisons. NHP, PH and JL wrote the paper.
